# MicroRNAs as Innovative Biomarkers for Inflammatory Bowel Disease and Prediction of Colorectal Cancer

**DOI:** 10.3390/ijms23147991

**Published:** 2022-07-20

**Authors:** Letizia Masi, Ivan Capobianco, Carlotta Magrì, Irene Marafini, Valentina Petito, Franco Scaldaferri

**Affiliations:** 1 Centro per le Malattie dell’Apparato Digerente (CEMAD), Dipartimento di Scienze Mediche e Chirurgiche, Fondazione Policlinico Universitario “A. Gemelli” IRCCS, Largo A. Gemelli 8, 00168 Roma, Italy; letizia.masi@guest.policlinicogemelli.it (L.M.); franco.scaldaferri@unicatt.it (F.S.); 2 Dipartimento Universitario di Medicina e Chirurgia Traslazionale, Università Cattolica del Sacro Cuore, Largo F. Vito 1, 00168 Roma, Italy; ivan.capobianco01@icatt.it (I.C.); carlotta.magri01@icatt.it (C.M.); 3 UOC Gastroenterologia ed Endoscopia Digestiva, Dipartimento di Medicina, Fondazione Policlinico Tor Vergata, Viale Oxford 81, 00133 Roma, Italy; irene.marafini@gmail.com

**Keywords:** microRNAs (miRNAs), inflammatory bowel disease (IBD), colorectal cancer (CRC), biomarkers

## Abstract

Inflammatory bowel disease (IBD) includes ulcerative colitis (UC) and Crohn’s disease (CD). These are autoimmune diseases of the gastrointestinal tract with a chronic relapsing and remitting course. Due to complex interactions between multiple factors in the etiology of IBD, the discovery of new predictors of disease course and response to therapy, and the development of effective therapies is a significant challenge. The dysregulation of microRNAs (miRNAs), a class of conserved endogenous, small non-coding RNA molecules with a length of 18–25 nucleotides, that regulate gene expression by an RNA interference process, is implicated in the complex pathogenetic context of IBD. Both tissue-derived, circulating, and fecal microRNAs have been explored as promising biomarkers in the diagnosis and the prognosis of disease severity of IBD. In this review, we summarize the expressed miRNA profile in blood, mucosal tissue, and stool and highlight the role of miRNAs as biomarkers with potential diagnostic and therapeutic applications in ulcerative colitis and Crohn’s disease. Moreover, we discuss the new perspectives in developing a new screening model for the detection of colorectal cancer (CRC) based on fecal miRNAs.

## 1. Introduction

Inflammatory bowel disease (IBD) refers to a family of inflammatory diseases of the digestive tract with a chronic relapsing and remitting course. IBD comprises two main forms—Crohn’s disease (CD) and ulcerative colitis (UC). The highest incidence rate of IBD is at a young age, equally between genders. The frequency of this condition is increasing dramatically around the world, with an evident increase in developing countries and young children [1]. These disorders of chronic intestinal inflammation are thought to develop primarily in genetically predisposed subjects in association with a microbial dysbiosis, dysregulated response, and environmental triggers (e.g., psychosocial stress, diet, smoking) [2,3,4].

In UC, only the colonic mucosa is inflamed, generally ranging from the rectum with extension towards the cecum; the main symptoms include rectal tenesmus, bleeding, diarrhea, abdominal pain, and fecal incontinence. In CD, inflammation can involve any part of the gastrointestinal (GI) tract from the mouth to the anus. The terminal ileum and colon are the most affected and abdominal pain, chronic diarrhea, and weight loss are common symptoms of CD [5,6]. First-line therapy for CD include immunosuppressants (e.g., azathioprine, mercaptopurine, and methotrexate), corticosteroids, anti-TNF therapy (e.g., infliximab, golimumab, adalimumab), monoclonal antibodies (e.g., the anti-alpha4beta7 integrin inhibitor vedolizumab, the anti-IL-12/IL-23p40 antibody ustekinumab), and surgery for unresponsive patients. Preferred therapeutic agents for UC include 5-aminosalicylic acid, corticosteroids, anti-TNF agents (e.g., infliximab, golimumab, adalimumab), monoclonal antibodies (e.g., vedolizumab and ustekinumab), newer target therapies (the JAK-inhibitor tofacitinib, the sphingosine-1-phosphate receptor modulator ozanimod) [7,8,9,10,11].

**Figure 1:**
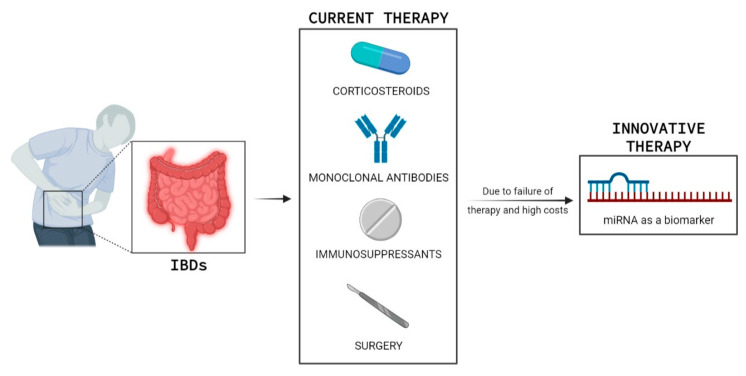
Inflammatory bowel disease (IBD) is a chronic invalidating disorder. Current medications (corticosteroids, monoclonal antibodies, immunosuppressants, and surgery) are associated with high costs, adverse events, and low response rates in the long term, hence the need to search for new biomarkers of response to therapy such as miRNA. This figure was created with BioRender. Available online: https://biorender.com.

Since IBD has become a worldwide-spread disease, the necessity of innovative therapeutic strategies has peaked. This is emphasized by the fact that currently used therapies are usually associated with high costs, adverse events, and low response rates in the long run; in addition, there are few indicators to help the clinician in selecting therapies [12,13]. New molecular predictors of response to therapy, new targets for innovative therapies, and new understandings within the complex pathogenesis of IBD are the present clinical needs and the main goal of scientific research (Figure 1).

MicroRNAs (miRNAs) are non-coding single-stranded RNAs of 18–25 nucleotides derived from primary miRNA transcripts (pre-miRNA) of intergenic or intronic origin. Recently, several papers have focused their investigation on the altered expression of miRNAs in IBD and their important role as regulators and possible diagnostic biomarkers in IBD. MicroRNAs do not seem to only be interesting tools for diagnosis, but also have a potential future therapeutic application that relies on miRNA mimics or miRNA antagonists [6,14,15].

## 2. MicroRNAs–Biogenesis and Function

MicroRNAs (miRNAs) are a class of endogenous, small non-coding RNA molecules that regulate organic phenomenon by RNA interference processes, including mRNA chopping, mRNA deadenylation, and translation inhibition.

The biogenesis of microRNAs begins within the nucleus where RNA polymerase II transcribes miR genes, generating a primary miRNA (pre-miRNA), which is subsequently cleaved by Drosha (RNase III endonuclease), leading to a precursor miRNA (pre-miRNA) [16]. The pre-miRNA is cleaved by the cytoplasmic endonuclease Dicer forming a duplex miRNA complex [17]. The active strand of the resulting miRNA duplex, of roughly 22 nucleotides long, is incorporated into the RNA-induced silencing complex (RISC), a multi-subunit complex composed of Argonaute proteins. The miRNA-loaded RISC (miRISC) is now competent to induce gene silencing because the loaded miRNA provides specificity by binding to complementary sequences on the target miRNA 3′ untranslated region (UTR) [18]. The regulatory activity of miRNAs typically involves the translational repression of target mRNAs or the decrease in mRNA stability, leading to a discount of the ultimate protein output from a given mRNA transcript. With the advent of miRNA arrays and high-throughput RNA sequencing techniques, it has been observed that miRNAs not only circulate within human peripheral blood in an exceedingly stable form, but they are also present in other bodily fluids and feces [19].

The role of miRNAs in various cellular processes has been established, including cell division and death, cellular development, proliferation, replicative senescence, intracellular signaling, and aging. It is now clear that dysregulated expression of miRNAs can deeply impact cell function and, as a result, is involved in various pathological, and sometimes malignant, conditions [20]. MiRNA expression has been shown to be of importance in a vast selection of human diseases such as cancer, autoimmune, cardiovascular, and neurodegenerative diseases [21,22,23].

## 3. MiRNAs in IBD

In IBD, miRNAs are found to be involved in pathogenesis and are identified as both diagnostic biomarkers and therapeutic targets. Most of the recent research within the IBD field has measured the levels of circulating miRNAs in body fluids such as blood or feces, and in homogenized tissue and biopsies using techniques such as microarray profiling, RT-qPCR, and NGS [24,25]. MiRNAs play important roles in IBD onset, either inhibiting or enhancing immune and inflammation signals by regulating the expression of the positive or negative components of immune signaling pathways related to IBD and CRC progression.

## 4. Mucosal Tissue miRNAs in IBD

Several studies have examined miRNA expression profiles in tissue derived from IBD patients [26].

In 2008, Wu et al. found that miRNAs regulate colonic epithelial-cell-derived chemokine expression; this was the first study to demonstrate the link between miRNAs and IBD. They found that miR-16, miR-21, miR-23a, miR-24, miR-29a, miR-126, miR-195, and let-7f were upregulated in active UC patients compared to healthy controls, while decreased levels of miR-188-5p, miR-215, miR-320a, and miR-346 were observed within the inflamed mucosa of UC patients [27]. By comparing the colonic mucosa of UC patients and healthy controls, miR-7, miR-26a, miR-29a, miR-29b, miR-31, miR-126* (the complement of miR-126), miR-127-3p, miR-135b, and miR-324-3p were increased within the inflamed mucosa of UC patients [28]. The mucosal tissue samples of patients with active CD had aberrant expression of miRNAs compared to controls. They also examined eight patients with CD compared with 10 healthy controls and identified 23 miRNAs significantly upregulated in active CD tissues (miRs-9, -21, -22, -26a, -29a, -29c, -30b, -31, -34c-5p, -106a, -126, -126*, -127-3p, -130a, -133b, -146a, -146-3p, -150, -155, 181c, -196a, -324-3p, -375). Five of those miRNAs were specific for active CD (miRs-9, 126, -130a, -181c and -375), while the remaining 18 were also upregulated in quiescent CD tissue [28].

MicroRNAs such as miR-141, miR-200a, miR-200b, miR-200c, and miR-429 were found to be significantly down-regulated in CD compared to the traditional or the smallest amount of affected mucosa [29]; three of those microRNAs (miR-141, miR-200b, and miR-429) were also found in UC.

The expression of miR-146a and -155 was higher in the inflamed mucosa of young patients with CD and UC than in the intact mucosa [30]. Mir-21, miR-155, and miR-31 are the foremost relevant IBD-associated miRNAs; miR21 is possibly the most intriguing miRNA involved in IBD, with associations between miR21 and IBD being observed in several studies, as well as functional relevance reported in mouse models of IBD [6,31,32].

Recently, the role of miRNAs in CD had been investigated by studying the location-specific differential expression of miRNAs in the tissue of inactive or minimally inflamed CD subjects. Mohammadi et al., found a panel of nine miRNAs whose expression pattern was driven by both diagnosis and site of biopsy: miR-215-5p, miR-203a-3p, miR-223-3p, miR-194-5p, miR-192-5p, miR-10b-5p, miR-10a-5p, miR-337-5p, and miR-582-5p [33]. Furthermore, miR-223, which showed an age-related effect in their study, has previously been shown to suppress NLRP3, [34,35] an activator of NF-kappaB signaling and a key component within the inflammatory response. A recent study [36] even showed that synthetic mimics of miR-223, delivered via nanoparticles, reduced levels of NLRP3 and inflammation during a murine model of experimental colitis, suggesting the possibility of miRNA-based therapeutics targeting inflammation within the gut.

A series of IBD- and CRC-related tissue miRNA studies from recent years are shown in Table 1.

## 5. Circulating miRNAs in IBD

The majority of studies on the altered expression of miRNAs in IBD are conducted in tissue and cell cultures, and there are currently few reports on the quantitative assessment of circulating miRNAs in IBD patients. The most used techniques to review circulating miRNAs are an miRNA microarray and miRNA quantitative RT-PCR.

With those techniques, five miRNAs (miR-199a-5p, miR-362-3p, miR-340*, miRplus-E1271, and miR-532-3p) were found significantly increased and two miRNAs (miR-149* and miRplus-F1065) were significantly decreased within the blood of active CD patients, as compared to healthy controls. Twelve miRNAs (including miR-28-5p, miR-151-5p, miR-103-2*, miR-199a-5p, miR-340*, miR-362-3p, miR-532-3p, miR-505*, and miRplus-E1271) were drastically increased and miRNA-505 was significantly decreased within the blood of active UC patients, as compared to healthy controls. Ten miRNAs (including miR-28-5p, miR-103-2*, miR-149*, miR-151-5p, miR-340*, miR-532-3p, and miR-plus-E1153) were significantly increased, and one miRNA (miRNA-505*) was considerably decreased within the blood of active UC patients, as compared to active CD patients [37].

Using reverse transcription and real-time PCR to quantitatively asses the miRNA expression patterns, MiR-16, miR-23a, miR-29a, miR-106a, miR-107, miR-126, miR-191, miR-199a-5p, miR-200c, miR-362-3p, and miR-532-3p were found. They were expressed at significantly higher levels within the blood from patients with CD compared with the healthy controls; within the UC cases, miR-16, miR-21, miR-28-5p, miR-151-5p, miR-155, and miR-199a-5p were significantly increased compared to healthy controls [38].

Specific miRNA expression patterns within the serum of IBD patients were studied, and it had been found that not only the amount of serum miRNAs in CD and UC were different to healthy subjects, but they were also different in numerous stages of IBD. Six miRNAs (miR-188-5p, miR-877, miR-140-5p, miR145. miR-18a, and miR-128) expressed differentially within the serum of active CD patients, compared with inactive CD patients, are identified [39].

Chen P. et al. [40] demonstrated that serum microRNA146b-5p (miR-146b-5p) expression was 2.87- and 2.72-fold higher in patients with regional ileitis and inflammatory bowel disease, respectively, than in healthy controls. Serum miR-146b-5p was significantly correlated with disease activity and was more specific than serum globulin (CRP). Sera samples from IBD patients showed a higher level of miR-16, miR-21, and miR-223, compared to controls, and was higher in CD than in UC patients [41].

Recently, it had been found that miR-21-5p was downregulated within the sera and colon tissue of UC compared with healthy people, and therefore the control group [42].

Circulating miRNAs could also be used to monitor the disease activity in patients with IBD, to predict the course of the disease, and to tell IBD apart from infectious colitis. For instance, it was found that miR-320a expression in the peripheral blood from patients with IBD follows the clinical and endoscopic disease activities: in comparison with healthy controls, miR-320a blood levels were significantly increased in patients with active CD and UC, and patients with inactive IBD. In both patients with CD and UC, miR-320a levels showed a robust correlation with the endoscopic disease activity. Finally, miR-320a blood expression in patients with active CD and UC significantly increased compared with patients with infectious colitis [43].

A series of IBD- and CRC-related circulating miRNA studies from recent years are shown in Table 2.

## 6. Fecal miRNAs in IBD and as Screening for Colorectal Cancer (CRC)

MiRNAs are present in all body fluids, including stools, with different compositions and concentrations. Fecal miRNAs were first observed in 2008 [44] and several other subsequent studies revealed that they are altered in many intestinal diseases, mainly in IBD and CRC. Recently, it has been discovered that fecal miRNAs influence the gut microbiota [20,45].

In CD and UC patients, feces samples are also better linked to expression levels within the diseased mucosa than to the degree within the blood circulation. Fecal miRNAs differ between IBD patients and healthy subjects and might represent potential non-invasive biomarkers for IBD. Verdier et al. [46] analyzed fecal miRNAs in IBD and located increased levels of miR-223 and miR-1246 within the stool of active IBD patients versus the control group. Another study found that miR-223 is upregulated within the feces of patients with IBD [47]. Additionally, the authors found that fecal levels of miR16 and miR-223 correlated with clinical parameters, such as C-reactive protein and calprotectin. MiR-16-5p was up-regulated in UC and CD patients, and miR-21-5p was up-regulated in UC patients, compared with healthy controls [48].

MiRNAs also can regulate the uptake of bacterial products to affect the intestinal pathology of the host. For instance, miR-193a-3p was reported to affect the absorption of bacterial products to ameliorate DSS-induced colonic inflammation by targeting PepT1, which helped to soak up bacterial products, and its expression increased in colonic tissues with inflammation [49]. Fecal miRNAs may be obsessed by the gut microbiota to vary the expression of their genes and composition. As an example, miR-515-5p elevated the proportion of Fusobacterium nucleatum 16S rRNA/23S rRNA transcripts, and miR-1226-5p upregulated the extent of yegH mRNA in E. coli. What is more, these two miRNAs promoted the expansion of Fusobacterium nucleatum (Fn) and E.coli, which have been identified to drive CRC [45].

MiRNAs are believed to play a part in the inflammation in IBD and to be implicated within the process of moving from inflammation to CRC [50]. It is now understood that miRNA dysregulation may be a central event within the development and pathophysiology of several cancers. MiRNAs likely play both oncogenic and tumor-suppressive roles within the carcinogenesis and progression of CRC by regulating the expression of diverse cancer-related genes.

In a case-control trial, it was reported that feces from patients with CRC expressed miRNA-21 and miRNA-106a increased levels [51]. Ahmed et al. detected the miRNA expression within the stool of CRC patients and identified the increased expression of seven miRNAs including miR-21, miR-106a, miR-96, miR-203, miR-20a, miR-326, and miR-92 and reduced expression of seven other miRNAs, miR-320, miR-126, miR-484-5p, miR-143, miR145, miR-16, and miR-125b, within the stool of CRC patients [44].

Recently, a fecal miRNA-based algorithm has been proposed for the screening for CRC. Sanchon et al. [52] found that miR-421, miR130b-3p, and miR27a-3P were upregulated in fecal samples from patients with CRC. The authors developed a model, within which the mix of the fecal level of MIR421, MIR27a-3p, and hemoglobin identified patients with CRC with an area under the curve (AUC) of 0.93, compared with an AUC of 0.67 for fecal hemoglobin concentration alone. In addition, they identified an algorithm which supported fecal levels of two microRNAs (miR-421 and miR-27a-3p), fecal hemoglobin concentration, and patient age and sex identifying patients with advanced colorectal neoplasia [53]. The results show that patients with advanced neoplasm are often identified through an miRNA signature in feces, the accuracy of which seems to be superior to that achieved with fecal hemoglobin concentration in fecal immunochemical test [FIT]–positive individuals. Including fecal miRNAs in fecal immunochemical-test-based colorectal cancer screening could increase their effectiveness and efficiency and this might represent another fascinating potential role of fecal miRNAs. 

A series of IBD- and CRC-related fecal miRNA studies from recent years are shown in Table 3.

## 7. MiRNAs as Biomarkers, and Potential Diagnostic and Therapeutic Applications

In recent years, extraordinary progress has been made in terms of identifying the origin and exact functions of miRNA, specializing in their potential use in both research and also the clinical field.

The main circulating miRNA sources are extracellular vesicles that are shed into the blood from many cell types. The association of miRNAs with proteins and their small size prevents degradation by RNases and enhances their stability in blood, making them an honest candidate in biomarker research. However, extraction and analysis of miRNAs from these extracellular vesicles may present challenges [54].

In addition to the potential role as diagnostic biomarkers discussed above, miRNAs in IBD are also interesting tools for potential future therapeutic applications (Figure 1).The goal of the treatment of IBD patients is to get remission and mucosal healing.

Biologic therapies directed against inflammatory mediators involved within the pathophysiology of IBD have revolutionized medical treatment: anti-TNF therapy (e.g., infliximab, golimumab, adalimumab), monoclonal antibodies (e.g., the anti-alpha4beta7 integrin inhibitor vedolizumab and the anti-IL-12/IL-23p40 antibody ustekinumab), newer target therapies (the JAK-inhibitor tofacitinib, and the sphingosine-1-phosphate receptor modulator ozanimod) [7,8,9,10,11].

While these biologics have the advantage of specificity, as compared to glucocorticoids and other immunosuppressive agents (azathioprine, methotrexate), not all patients respond to these medications, and a few encounter significant side effects. Thus, miRNA-based therapeutics could represent a major alternative to those biologics. The previously referenced studies have clearly shown that miRNA deregulation is prevalent in IBD patients. The next logical step is to repair the erroneous miRNA expression by a therapeutic increase or decrease, reckoning on the kind of dysregulation.

The potential of developing miRNAs to focus on IBD-associated genes is intriguing. However, several limitations have yet to be solved in terms of miRNA-based therapeutics. To achieve success, miRNA modulators must be specific, efficient, and safely deliverable to the affected tissue. Off-target side effects are still a significant concern as altering the function of one miRNA could affect many downstream gene targets and pathways. Site-specific delivery of miRNA therapeutics remains challenging [26].

**Figure 2:**
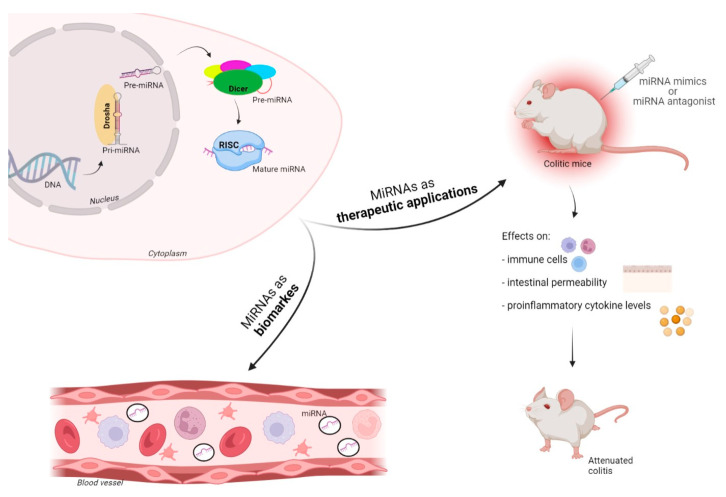
MiRNAs may play a role both as diagnostic biomarkers and in therapy: miRNA-based therapies comprise miRNA mimics or miRNA antagonists. This figure was created with BioRender. Available online: https://biorender.com.

MiRNA-based therapies comprise two fundamental strategies: miRNA mimics or miRNA antagonists (Figure 2). Physiologic miRNA over-expression leading to pathologically reduced target organic phenomenon is hindered by using miRNA antagonists, while reduced miRNA expression leading to enhanced target function is restored by utilizing miRNA mimics [6,55]. Several miRNA-based therapeutic studies have reported positive leads in animal models of IBD. The miR-301a expression levels were elevated during a TNBS-induced mouse colitis model compared with control mice; similarly, this occurred in PBMCs and inflamed mucosal IBD tissues. Mechanistic studies revealed that miR-301a promotes Th17 cell differentiation through direct regulation of Smad nuclear interacting p 1. Furthermore, enema administration of an miR-301a inhibitor in TNBS-induced mice reduced the number of proinflammatory cytokines within the inflamed colon [56]. Inverse correlation between miR-124 and aryl hydrocarbon receptor (AHR) protein levels have been observed in colon tissues and intestinal epithelial cells of active CD patients. Notably, anti-miR-124 treatment of TNBS-induced colitis mice improved colitis scores, including a decreased disease activity index and proinflammatory cytokine expression, through AHR modulation [57].

Anti-miR inhibition of miR-30c and miR-130a reduced intestinal inflammation during a mouse ileal loop model [58]. In IL-10 knockout mice and TNBS models of colitis, intestinal inflammation was exacerbated or reduced after intracolonic administration of an anti-miR (for inhibition) or pre-miR miRNA mimic (for overexpression) to miR-141, respectively [59].

A recent study [60] reported that a so-called antagomir towards miR-155 alleviated DSS-induced intestinal inflammation in mice and, therefore, the authors propose that anti-miR-155 can be a promising candidate for a unique IBD therapy. Interestingly, miR-155 has been demonstrated to control the expression of tight-junction proteins occludin, claudin-1, and MLCK [61]. Colitis is regulated by miR-133α-afthiphilin direct interaction; intracolonic administration of antisense-miR-133α before induction of TNBS- and DSS-induced colitis resulted in attenuation of colitis and inhibition of pro-inflammatory signals [62].

A study conducted on miR-133a and its target UCP2 (mitochondrial uncoupling protein 2) using the DSS-induced IBD mouse model found that miR-133a levels were decreased upon DSS treatment, and by introducing an miR-133a mimic, the DSS-induced IBD was alleviated, suggesting that miRNA mimics could also function as therapy in IBD [63]. MiR320a was shown to be abnormally expressed in mucosal biopsies from IBD patients; also, the role of IL-33-dependent regulation of miR320a during IBD continues to be unknown [28]. Results of a piece of work published in 2018 indicate that mice IL-33 induces increased epithelial miR-320 expression in immunocompetent BL6, which promotes epithelial repair/restitution and confers normal mucosal healing [64]. MiRNAs are a promising area of research for IBD therapeutics. Assessing the relevance of in vitro studies with functional research studies in vivo is critical to support the clinical translation of miRNA-based therapeutic approaches [65].

Interestingly, nine miRNAs, along with five clinical factors, correlated with response to treatment of IBD patients, and neural-network-developed algorithms supported certain miRNA levels identifying responders to the anti-TNF antibody therapy, infliximab, vs. non-responders [66].In a recent study [67], five candidate miRNAs were identified related to clinical response and mucosal inflammation in pediatric IBD patients (miR-126, let-7c, miR-146a, miR-146b, and miR-320a) and, therefore, the authors propose that these miRNAs could also be further developed as pharmacodynamic and response-monitoring biomarkers to be used in clinical care and trials.

## 8. Future of miRNAs in the Clinic

MiRNAs are attracting a growing interest in the scientific community due to their central role in the etiology of major diseases. As mentioned above, miRNA mimics and miRNA antagonists that restore miRNA expression or downregulate aberrantly expressed miRNAs, respectively, are highly sought-after therapeutic strategies for effective manipulation of miRNA levels. In this regard, carrier vehicles that facilitate the proficient and safe delivery of miRNA-based therapeutics are fundamental to the clinical success of these pharmaceuticals. 

A recent study [68] showed that systemic administration of miR-4689 mixed with the nanoparticles in nude mice inhibited the growth of mutant KRAS CRC cell line (DLD1) xenografts compared to miRNA negative control oligonucleotide administration. An artificial small RNA sequence (MIRTX) is found to have a potential therapeutic strategy in KRAS-mutant colorectal cancer [69]. In this work the researchers demonstrated that the administration of super carbonate apatite (sCA) nanoparticles–MIRTX conjugates in a CRC mouse model could suppress NF-kB signaling pathways via direct inhibition of the expression two proteins (CXCR2 and PIK3R1).

These results may open up new possibilities for an miRNA targeted therapy against intractable KRAS CRC. 

**Figure 3:**
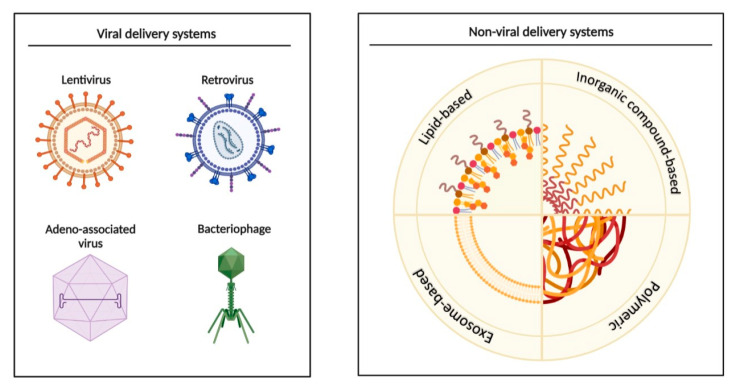
Viral and non-viral miRNA delivery systems. Viral methods represented here include lentivirus, retrovirus, adeno-associated virus and bacteriophage, while lipid-, inorganic-, exosome-, and polymeric based delivery are part of non-viral methods. This figure was created with BioRender. Available online: https://biorender.com.

MicroRNA delivery systems are tested for a variety of pathologies. The use of a peptide/miR-31 nanomedicine within an electrospun biomaterial promotes enhanced wound repair through the targeting of both stromal and epithelial compartments, both in vitro (HaCaT keratinocyte and HMEC-1 endothelial cells) and in vivo (C57BL/6J mice) [70]. A study conducted on ovarian cancer mice showed that miR-199a-3p incorporated into exosomes drastically inhibited peritoneal dissemination [71], suggesting therapeutic features of engineered exosomes. In addition to nanoparticles, there are also viral delivery systems that can be adapted for specific transgenes, treatment purposes, and targeted cell types (Figure 3). In fact, recombinant adeno-associated virus (rAAV) was used to evaluate the efficacy of the combined use of miR-26a and miRNA-122 on Huh7-cell-induced live tumors in a murine xenograft model in vivo, offering a potentially useful approach to target human liver tumors [72]. In another study [73], rAAV was capable of delivering miRNA-298 at a neuronal level, resulting in efficient transduction of muscle and motor neurons, down-regulation of androgen receptor expression, and amelioration of the disease phenotype in spinal and bulbar muscular atrophy mice.

The first miRNA molecule that is being evaluated in a clinical trial is miravirsen. The drug is under phase II trials for the treatment of hepatitis C viral infection [74], undergoing assessment for its safety and effectiveness in patients. To date, several miRNA molecules are in clinical trials but none have entered phase III. For this reason, academic laboratories, biotech companies, and the pharmaceutical industry are all involved in the clinical research efforts.

## 9. A Step Forward towards Personalized Medicine

As has been observed over the past several years, for human DNA and for gut microbiota sequencing, in the beginning it is always difficult to move from the research field to clinical practice, especially in relation to the cost of the methodologies. The first step will be to construct regulatory networks based on Gene Ontology annotation and the KEGG pathway of miRNAs from blood, tissue, and feces, explored through microarray analysis. This kind of analysis will provide the specific miRNAs involving in modulating crucial steps of the immune response, and/or in impaired epithelial barrier function. The costs of all these steps are high, especially the cost of bioinformatic analysis. In 2012, Keith W. Jones’s research group tried this approach and showed that circulating miRNAs segregate into four highly correlated intensity clusters [75]. Only after these types of studies will it be possible to recognize a cluster of a few important miRNAs. They will also be identified by qPCR, a more economic approach, that can be used in every laboratory, in every hospital. MicroRNAs can be recognized in different types of samples (blood, feces, and homogenized tissue biopsies) using qPCR, microarray profiling, NGS, and in situ hybridization (ISH) [24,25,76,77]. ISH is used to determine tissue miRNA expression, and it offers the chance to understand the cellular origin of miRNA and also the diseases mechanism involved. Obtaining tissue samples, on the other hand, requires an invasive procedure. Circulating miRNAs may derive from the tissue, but the study by Iborra et al. [39] showed that none of the serum miRNAs corresponded with tissue miRNAs in IBD patients. Otherwise, Schönauen et al. [41] found increased levels of miR-16, miR-21, and miR-223 in both sera and feces in CD and UC patients. Furthermore, miR-16 and miR-223 from feces correlated with clinical parameters, such as C-reactive protein and calprotectin, showing fecal samples to be a promising alternative to the expression levels in the mucosa.

Another important challenge in IBD treatment is to find a marker able to predict the response to therapy, especially to biological drugs. Nine miRNAs, together with five clinical factors, correlated with response to the anti-TNF antibody therapy infliximab, vs. non-responders [66]. Currently, therapies in IBD also include a monoclonal antibody against IL-12 and IL-23 (ustekinumab), and a monoclonal antibody against α4β7-integrin on T-helper cells (vedolizumab). The levels of miR-29, and its effect on the reduction in IL-23 expression, could potentially be a parameter of the efficacy of therapy with ustekinumab [55]. The same potential exists for miR-126 and vedolizumab; Harris et al. [78] described how endogenous miR-126 inhibits leukocyte adherence through the regulation of VCAM-1, an adhesion molecule expressed by endothelial cells.

## 10. Conclusions

The role of miRNAs in inflammation is well established and interesting. MiRNAs could potentially play an intriguing role in terms of settling on the most effective treatment strategy in line with the microbiota and immunological signatures. With the recent FDA approval of four siRNA-based drugs, the potential of RNA therapeutics has become a reality [79].

The search for biomarkers is an urgent clinical need, and miRNAs appear to be promising tools to move towards personalized medicine, especially regarding the response to biological therapies.

## Figures and Tables

**Table 1:** A summary of studies on mucosal tissue microRNA research in inflammatory bowel disease (IBD). CD: Crohn’s disease, UC: Ulcerative colitis, HC: Healthy controls, Biopsy: Colon tissue biopsy.

	MiRNAs	Disease Subtype	Sample Type	Results	Reference
1	miR-16, miR-21, miR-23a, miR-24, miR-29a, miR-126, miR-195, let-7f	UC, HC	Biopsy	These miRNAs were upregulated in active UC patients compared to healthy controls	[27]
2	miR-188-5p, miR-215, miR-320a, and miR-346	UC, HC	Biopsy	These miRNAs were downregulated in active UC patients compared to healthy controls	[27]
3	miR-7, miR-26a, miR-29a, miR-29b, miR-31, miR-126*, miR-127-3p, miR-135b, and miR-324-3p	UC, HC	Biopsy	These miRNAs were upregulated in active UC patients compared to healthy controls	[28]
4	miRs-9, -21, -22, -26a, -29a, -29c, -30b, -31, -34c-5p, -106a, -126, -126*, -127-3p, -130a, -133b, -146a, -146-3p, -150, -155, 181c, -196a, -324-3p, -375	CD, HC	Biopsy	These miRNAs were upregulated in patients with Crohn’s colitis patients compared to healthy controls	[28]
5	miR-141, miR-200a, miR-200b, miR-200c and miR-429	CD, HC	Biopsy	These miRNAs were downregulated in CD patients compared to healthy controls	[29]
6	miR-141, miR-200b and miR-429	UC, HC	Biopsy	These miRNAs were downregulated in active UC patients compared to healthy controls	[29]
7	miR-146a and -155	UC, HC	Biopsy	miR-146a and -155 was higher in the inflamed mucosa of children with CD and UC than in the intact mucosa	[30]
8	miR-215-5p, miR-203a-3p, miR-223-3p, miR-194-5p, miR-192-5p, miR-10b-5p, miR-10a-5p, miR-337-5p, miR-582-5p	CD, HC	Biopsy	Nine miRNAs were differentially expressed across HC and CD, accounting for biopsy location	[33]

**Table 2:** A summary of studies on circulating microRNA research in inflammatory bowel disease (IBD). CD: Crohn’s disease, UC: Ulcerative colitis, HC: Healthy controls.

	MiRNAs	Disease Subtype	Sample Type	Results	Reference
1	miR-199a-5p, miR-362-3p, miR-340*, miRplus-E1271, miR-532-3p	CD, HC	Blood	These five miRNAs were significantly increased in active CD patients, as compared to healthy controls	[37]
2	miR-149* and miRplus-F1065	CD, HC	Blood	These two miRNAs were significantly increased in active CD patients, as compared to healthy controls	[37]
3	miR-28-5p, miR-151-5p, miR-103-2*, miR-199a-5p, miR-340*, miR-362-3p, miR-532-3p, miR-505*, miRplus-E1271	UC, HC	Blood	These twelve were significantly increased in active UC patients, as compared to healthy controls	[37]
4	miRNA-505	UC, HC	Blood	miRNA-505 was significantly decreased in active UC patients, as compared to healthy controls	[37]
5	MiR-16, miR-23a, miR-29a, miR-106a, miR-107, miR-126, miR-191, miR-199a-5p, miR-200c, miR-362-3p and miR-532-3p	CD, HC	Blood	These miRNAs were significantly increased compared to healthy controls	[38]
6	miR-16, miR-21, miR-28-5p, miR-151-5p, miR-155 and miR-199a-5p	UC, HC	Blood	These miRNAs were significantly increased compared to healthy controls	[38]
7	miR-188-5p, miR-877, miR-140-5p, miR145. miR-18a, miR-128	CD	Serum	Six miRNAs expressed differentially in active CD patients compared with inactive CD patients	[39]
8	miR-146b-5p	CD, UC, HC	Serum	miR-146b-5p expression was higher in patients with CD and UC, than in healthy controls.	[40]
9	miR-16, miR-21, miR-155, and miR-223	CD, UC, HC	Serum, Feces	These miRNAs were significantly increased compared to healthy controls, and was higher in CD than in UC patients	[41]
10	miR-21-5p	UC, HC	Serum, Biopsy	miR-21-5p was downregulated in UC patients compared with healthy people and the control group	[42]
11	miR-320a	CD, UC, HC	Blood	miR-320a expression in patients with IBD follows the clinical and endoscopic disease activities	[43]

**Table 3:** A summary of studies on fecal microRNA research in inflammatory bowel disease (IBD) and colorectal cancer (CRC). CD: Crohn’s disease, UC: Ulcerative colitis, HC: Healthy controls.

	MiRNAs	Disease Subtype	Sample Type	Results	Reference
1	miR-223 and miR-1246	CD, UC	Feces	miR-223 and miR-1246 was increased in active IBD patients versus the control group	[46]
2	miR-16-5p and miR-21-5p	CD, UC, HC	Feces	miR-16-5p was up-regulated in UC and CD patients and miR-21-5p was up-regulated in UC patients, compared with healthy controls	[48]
3	miRNA-21 and miRNA-106a	CRC, HC	Feces	miRNA-21 and miRNA-106a was increased in patients with CRC	[51]
4	miR-21, miR-106a, miR-96, miR-203, miR-20a, miR-326, and miR-92	CRC, HC	Feces	These miRNAs were increased in patients with CRC	[44]
5	miR-320, miR-126, miR-484-5p, miR-143, miR145, miR-16, and miR-125b	CRC, HC	Feces	These miRNAs were decreased in patients with CRC	[44]
6	miR-421, miR130b-3p miR27a-3P	CRC, HC	Feces	These miRNAs were upregulated in patients with CRC	[52]

## Data Availability

Not applicable.
